# Supplementary material for: The genome sequence of the Speckled Yellow, Pseudopanthera macularia (Linnaeus, 1758) (Lepidoptera: Geometridae)
Source: Wellcome Open Res. 2025 Aug 15;10:443. [Version 1] doi: 10.12688/wellcomeopenres.24673.1 (PMC12583914; doi:10.12688/wellcomeopenres.24673.1)
Supplement: Peer review history

# Reviewer report: Reviewer response for version 1

**DOI:** 10.21956/wellcomeopenres.27188.r129957

**Published:** 2025-11-03

**Authors:** Jose Martinez

**Affiliations:** 1 University of Florida, Florida, USA

The dataset has been constructed in accordance with established standards, with clear and accessible explanations that ensure transparency. The protocols and procedures employed are methodologically sound, and the accompanying documentation of methods and materials is presented in a manner that facilitates reproducibility. Owing to its high degree of standardization, comprehensive illustrations, and open accessibility, the dataset constitutes a valuable resource with broad applicability.

 Sanchez *et al.* present a comprehensive and meticulously assembled genome of the Speckled Yellow, *Pseudopanthera macularia* (Linnaeus, 1758), generated from sequencing a single female specimen. The assembly demonstrates exceptional completeness, as reflected by its high BUSCO score, and is organized into 32 chromosomal pseudomolecules, including both the W and Z sex chromosomes. Produced through rigorous methodological approaches, this work delivers a detailed and robust genomic resource.

 The authors emphasize the significance of this genomic information for future studies, particularly regarding the population structure of the species and intraspecific sex chromosome polymorphism. In addition, they provide valuable context by outlining the biological history of *P. macularia*. 

 Congratulations!

Are sufficient details of methods and materials provided to allow replication by others?

Yes

Is the rationale for creating the dataset(s) clearly described?

Yes

Are the datasets clearly presented in a useable and accessible format?

Yes

Are the protocols appropriate and is the work technically sound?

Yes

Reviewer Expertise:

Evolutionary Biology, Ecology, IPM, Integrative Biology, Entomology, Genomics

I confirm that I have read this submission and believe that I have an appropriate level of expertise to confirm that it is of an acceptable scientific standard.

# Reviewer report: Reviewer response for version 1

**DOI:** 10.21956/wellcomeopenres.27188.r129954

**Published:** 2025-10-11

**Authors:** Sivasankaran Kuppusamy

**Affiliations:** 1 Loyola College, Chennai, Tamil Nadu, India

Authors have done the chromosome-level genome assembly of *Pseudopanthera macularia* (Linnaeus, 1758). They sequence and assemble two haplotypes with the lengths of 717.87 Mb and 650.90 Mb respectively. Through the assembly they scaffolded into 32 chromosomes. Authors have used appropriate methodology and software for high molecular weight DNA isolation and sequencing, sequence assembly and annotations.

**Comments on the manuscript**

 Authors have given the species Linnaean hierarchy in the abstract. The species hierarchy may be removed in the abstract.  The total number of protein-coding genes, non-coding genes and gene transcripts can be included in the abstract. 

 The author used the full name of the genus, *Pseudopanthera macularia*, in the article. Start with the full genus name once, if possible, then shorten it to *P. macularia*.

 In the previous genome assemblies authors have detected protein-coding genes, non-coding genes and gene transcripts through the sequence assembly and annotations. In the present research authors haven’t given above mentioned details. Any reason?

 The article was well prepared and the manuscript meets the necessary scientific standard and is suitable for indexing.

Are sufficient details of methods and materials provided to allow replication by others?

Yes

Is the rationale for creating the dataset(s) clearly described?

Yes

Are the datasets clearly presented in a useable and accessible format?

Yes

Are the protocols appropriate and is the work technically sound?

Yes

Reviewer Expertise:

Phylogenetic analysis of Noctuoidea moths using mitogenome sequence

I confirm that I have read this submission and believe that I have an appropriate level of expertise to confirm that it is of an acceptable scientific standard.
